# Breast Cancer Incidence Rates in Ghanaian and US Black Women From 2013 Through 2015

**DOI:** 10.1001/jamanetworkopen.2025.37160

**Published:** 2025-10-13

**Authors:** Brittny C. Davis Lynn, Jonine D. Figueroa, Dennis Laryea, Fred Kwame Awittor, Naomi O. Ohene Oti, Quiera S. Booker, Lawrence Edusei, Nicolas Titiloye, Ernest Adjei, Beatrice Wiafe Addai, Robertson Adjei, Lucy T. Afriyie, Joel Yarney, Daniel Ansong, Seth Wiafe, Thomas Ahearn, Verna Vanderpuye, Florence Dedey, Louise A. Brinton, Baffour Awuah, Joe Nat Clegg-Lamptey, Mustapha Abubakar, Montserrat Garcia-Closas, Richard Biritwum, Barry I. Graubard

**Affiliations:** 1 Division of Cancer Epidemiology and Genetics, National Cancer Institute, Rockville, Maryland; 2 Usher Institute, Institute of Genetics and Cancer, University of Edinburgh, Edinburgh, Scotland; 3 Komfo Anokye Teaching Hospital, Kumasi, Ghana; 4 Korle Bu Teaching Hospital, Accra, Ghana; 5 Peace and Love Hospital, Kumasi, Ghana; 6 Ghana Statistical Service, Accra, Ghana; 7 Kwame Nkrumah University of Science and Technology, Kumasi, Ghana; 8 Loma Linda University, Loma Linda, California; 9 Cancer Epidemiology and Prevention Unit, Institute for Cancer Research and Imperial College, London, United Kingdom; 10 University of Ghana, Accra, Ghana

## Abstract

**Importance:**

Uncertainty exists about whether women of West African ancestry have increased rates of estrogen receptor (ER)-negative breast tumors, which commonly have earlier age onsets than ER-positive tumors and are aggressive. High-quality population-based studies in Africa with tumor pathology data are generally unavailable, and there is speculation that younger population age structures may explain the increased frequencies of reported ER-negative tumors.

**Objective:**

To estimate age-standardized and age-specific rates of ER-negative breast cancer among Ghanaian women and non-Hispanic Black and non-Hispanic White women in the US.

**Design, Setting, and Participants:**

This cross-sectional study used 2013 through 2015 data from a population-based case-control study conducted in 2 metropolitan areas of Ghana, Accra and Kumasi, to estimate ER-specific breast cancer incidence rates among women 18 to 74 years of age. Incidence rates were recovered by applying sampling weights for controls calculated by district, 5-year age groups, and nonresponse rates from the 2010 census-based enumeration area listings. US Breast Cancer Registry data were retrieved from 17 registries in the Surveillance, Epidemiology, and End Results (SEER) program for non-Hispanic Black and non-Hispanic White women with breast cancer 20 to 74 years of age. Analyses were conducted January 2020 through May 2025.

**Exposures:**

Age, sex, race and ethnicity, ER tumor status, and country (US and Ghana).

**Main Outcomes and Measures:**

For each of 3 populations, crude, age-standardized, and age-specific breast cancer incidence rates were calculated overall and by ER status.

**Results:**

Analyses were performed for 1071 women (age range, 18-74 years; 468 [51%] ER-negative) in Ghana and 18 321 non-Hispanic Black women (age range, 20-74 years; 5117 [29%] ER-negative) and 103 227 non-Hispanic White women (age range, 20-74 years; 15 040 [15%] ER-negative) in SEER. From 2013 through 2015, ER-negative age-standardized incidence rates were 42.3 (95% CI, 38.7-46.3) per 100 000 women for Ghanaian participants and 43.1 (95% CI, 42.0-44.3) per 100 000 for US non-Hispanic Black women, both higher rates than that of 24.0 (95% CI, 23.6-24.4) per 100 000 for US non-Hispanic White women. ER-positive age-standardized incidence rates were 42.1 (95% CI, 38.4-46.1) per 100 000 women for Ghanaian participants, 105.4 (95% CI, 103.6-107.3) per 100 000 for non-Hispanic Black women, and 128.5 (95% CI, 127.9-129.7) per 100 000 for non-Hispanic White women.

**Conclusions and Relevance:**

In this cross-sectional study of Ghanaian and US non-Hispanic Black and non-Hispanic White women with breast cancer, age-standardized tumor incidence rates for women with ER-negative tumors in Ghana were similar to those for US non-Hispanic Black women, and both rates were higher than for US non-Hispanic White women. This finding may suggest increased susceptibility requiring elucidation in US non-Hispanic Black and West African populations.

## Key Points

**Question:**

Do age-standardized incidence rates of estrogen receptor (ER)-negative and ER-positive breast tumors in Ghana support a possible increased susceptibility to ER-negative breast cancer in women of West African ancestry?

**Findings:**

This cross-sectional study including 1071 women in Ghana and 121 548 women in the US with breast cancer from 2013 through 2015 found that age-standardized incidence rates of ER-negative tumors were similar for Ghanaian and US non-Hispanic Black women. ER-positive tumors were lower for women from Ghana compared with non-Hispanic Black women.

**Meaning:**

Findings from this study of the similarity between Ghanaian and US non-Hispanic Black women for age-standardized incidence rates of ER-negative but not ER-positive breast tumors suggest that there may be shared etiologic factors associated with ER-negative breast cancer that need identification.

## Teaser

This cross-sectional study investigates age-standardized incidence rates of estrogen receptor–negative and estrogen receptor–positive breast tumors among Ghanaian women and US non-Hispanic Black and non-Hispanic White women with breast cancer.

## Introduction

Despite having historically low breast cancer incidence rates compared with many other countries,^1^ sub-Saharan African nations have experienced a rapid increase in incidence in more recent years.^2^ This increase in incidence has been attributed to changing risk factors, including delayed age at first infant birth and fewer children, as well as changes in diet, body weight, and other factors.^2^ Estrogen receptor (ER) status (positive or negative) is a fundamental characteristic of the epidemiology of breast cancer and a key marker for distinct etiology exhibited by different incidence trends and an important determinant of treatment options, disease outcomes, and survival.^3,4^ Due to limited resources, collection of ER status in cancer registries is currently not standard practice in many countries, including African nations. Understanding the frequency and rates of different subtypes of breast cancer in African populations is important to country ministries of health and policymakers to determine the burden of disease, drug, and treatment planning and whether receptor status testing should be standard to help in part to improve mortality outcomes—which are some of the worst worldwide.^5^ Understanding what factors contribute to different breast cancers in high-risk populations could also help to prevent the disease globally.

The etiology of breast cancer is complex, with genetic and nongenetic risk factors (eg, reproductive and lifestyle) whose associations differ by tumor subtypes.^3^ For example, a scoping review showed reproductive factors to have differential associations with breast cancer risk by ER status that were consistent in many populations.^3^ A comprehensive review by Eng et al^5^ showed considerable variation in the reported frequencies of ER status-defined tumors across studies on the African continent. That report concluded that the higher frequency of ER-negative tumors among African women could primarily reflect a much younger age distribution in the population and more advanced disease presentation due to the lack of screening for early detection.^5^

Age-standardized and age-specific incidence rates rather than proportions are necessary to conduct a more robust assessment of breast cancer incidence as well as to make comparisons across countries with different demographics. However, a lack of high-quality data on ER-specific incidence rates in many sub-Saharan African countries due to study design issues, such as unrepresentative case series and unstandardized tissue processing or ER staining, has been a major limitation.^5^

In the present report, using data from a high-quality epidemiologic study with population-based controls and high participation rates (over 90% for cases and controls), many prior limitations have been addressed, including obtaining quality ER status data to estimate age-standardized and age-specific breast cancer incidence rates in Ghana overall and by ER status. Rates between US non-Hispanic Black, non-Hispanic White, and Ghanaian women are compared herein to evaluate the plausibility of higher susceptibility to ER-negative tumors in populations with West African ancestry.

## Methods

This cross-sectional study used data from the Ghana Breast Health Study (GBHS) that has been previously described.^6,7,8^ The present study followed the Strengthening the Reporting of Observational Studies in Epidemiology (STROBE) reporting guideline. The GBHS was approved by the Special Studies Institutional Review Board of the National Cancer Institute (Rockville, Maryland), the Ghana Heath Service Ethical Review Committee, and the institutional review boards at the Noguchi Memorial Institute for Medical Research (Accra, Ghana), the Kwame Nkrumah University of Science and Technology (Kumasi, Ghana), the School of Medical Sciences at Komfo Anokye Teaching Hospital (Kumasi, Ghana), and Westat (Rockville, Maryland). All participants provided written informed consent. Briefly, the GBHS is a population-based case-control study that was conducted from 2013 through 2015 in 2 major cities in Ghana, Accra and Kumasi, that included women who presented with a breast mass and suspected breast cancer. The cases came from 3 major hospitals providing surgical and treatment options for breast cancer in Ghana for individuals who were living in catchment areas defined by 22 municipal districts and metropolitan areas in the Greater Accra, Central and Eastern (ie, Accra area), and Ashanti (ie, Kumasi area) regions and who could feasibly travel to the hospitals.^9^ The GBHS enrolled 2202 eligible women who presented with a breast mass at the time of biopsy and resided within catchment areas for at least 1 year. Of the 2202 enrolled cases, 1071 had a pathologically confirmed breast cancer diagnosis with known age, as previously described.^6^

The control participants were randomly sampled from the catchment areas using a stratified (stratifying on the 22 municipal districts and metropolitan areas) 2-stage cluster sample design with frequency matching by 5-year age groups (18-24, 25-29, 30-34, 35-39, 40-44, 45-49, 50-54, 55-59, 60-64, 65-69, and 70-74 years) determined at enumeration. Sample weights for controls were derived based on the inverse of the sampling fractions specified by the sample design (case sample weights were 1), nonresponse adjustments, and poststratification to the 2010 Ghana census. All estimates of crude, age-specific, and standardized incidence rates of breast cancer overall and by ER status and standard errors accounted for the sample weights and sample design using survey methods.^10^

Invasive breast cancer incidence rates for breast cancer overall as well as by ER status were obtained from 17 Surveillance, Epidemiology, and End Results (SEER) program registries for 2013 through 2015 for self-identified non-Hispanic Black and non-Hispanic White women 20 to 74 years of age. Data were limited to these groups as they represent the extremes in incidence of the major ER subtypes, with non-Hispanic White women having the highest ER-positive incidence rates, and non-Hispanic Black women have the highest ER-negative incidence rates and they likely share some ancestry with women from Ghana. The SEER program has established coding and historically provides 5 mutually exclusive race and ethnicity categories used for reporting cancer statistics defining non-Hispanic White and non-Hispanic Black.

The primary aim of the GBHS was to determine risk factors associated with different molecular pathologic subtypes of breast cancer. In a separate project,^11^ members of our team had 560 samples analyzed using nCounter (nanoString), which included a research version of the 50-gene PAM50 profile to classify tumor molecular subtypes: luminal A, luminal B, human epidermal growth factor receptor 2 (HER2)-enriched, basal-like, and normal-like. The ER status was inferred and classified in those samples as ER-positive when the PAM50 classifier determined them to be luminal A or luminal B, and ER-negative when the samples were HER2-enriched or basal-like. For samples not having nCounter-defined ER status, the study used the immunohistochemically (IHC)-derived ER status, which members of our team had previously shown to be of high agreement, beyond what was expected by chance (78% agreement observed, 50% expected).^8^ Based on 424 cases with both IHC and nCounter data, we found 79% agreement between the IHC and RNA expression–based method.

### Statistical Analysis

A single imputed ER status for each missing observation was computed separately for the GBHS and SEER by race and ethnicity and age at diagnosis.^12^ To calculate age-specific incidence rates of breast cancer in Ghana from the GBHS data, sample weights were applied to estimate the overall incidence rates in the population of women in Ghana. Age-specific incidence rates were calculated by taking the total number of invasive breast cancer cases from the GBHS and dividing by the weighted population-based controls. This ratio was divided by 3 years and multiplied by 100 000 persons to report the 3-year rate per 100 000 women in Ghana. Age-specific incidence by ER status was plotted using the population-level data with imputed ER status.^12^ Age-standardized incidence rates were estimated using the World Standard Segi 1960, which is used in International Agency for Research on Cancer Global Cancer Observatory data, to facilitate international comparisons.^13^ All GBHS analyses used R, version 4.3.2 (R Project for Statistical Computing). Analyses were conducted January 2020 through May 2025.

## Results

This study included 1071 Ghanaian women diagnosed with invasive breast cancer and 2106 women as weighted controls from the GBHS (age range, 18-74 years). From SEER, there were 18 321 invasive breast cancer cases among non-Hispanic Black women (age range, 20-74 years) and 103 227 among non-Hispanic White women (age range, 20-74 years) (Table 1). The highest percentage of Ghanaian women diagnosed with breast cancer was in the group 40 to 49 years of age (221 [30%]), followed by the group 50 to 59 years of age (281 [26%]), the group 60 years and older (253 [24%]), and the group younger than 40 years (221 [21%]) (Table 1). In contrast, non-Hispanic Black (7632 [42%]) and non-Hispanic White (53 774 [52%]) women had most breast cancers diagnosed in the groups 60 years of age and older. We aimed to obtain as much ER status data as possible on cases in Ghana, which resulted in only 13% missing ER status. In SEER, missing ER status was 4% for non-Hispanic Black women and 3% for non-Hispanic White women. ER-negative breast tumors (468 [51%]) were diagnosed at a slightly higher frequency compared with ER-positive breast tumors (458 [49%]) among Ghanaian women. Of 18 321 US non-Hispanic Black women, 5117 (29%) were ER-negative cases, and of 103 227 US non-Hispanic White women, 15 040 (15%) were ER-negative cases. The overall age-standardized breast cancer incidence rate for Ghanaian women from 2013 through 2015 was 84.4 (95% CI, 79.2-89.9) per 100 000 women. This rate was lower than that observed for non-Hispanic Black and non-Hispanic White women at 148.5 (95% CI, 146.4-150.7) and 152.9 (95% CI, 151.9-153.8) per 100 000 women, respectively (Table 2). Age and tumor characteristics for women diagnosed with breast cancer in GBHS and SEER are presented in Table 1, and crude and age-standardized incidence rates are presented in Table 2.

**Table 1.** Age and Tumor Characteristics for Women With Breast Cancer in Ghana (GBHS) and Non-Hispanic Black and Non-Hispanic White Women in the US (SEER 17), 2013-2015

Characteristic	Participants, No. (%)		
	GBHS	Non-Hispanic Black, SEER 17	Non-Hispanic White, SEER 17
Age range, y	18-74	20-74	20-74
No. of cases	1071	18 321	103 227
Age at diagnosis, y			
<40	221 (21)	1359 (7)	4459 (4)
40-49	316 (30)	3506 (19)	15 931 (15)
50-59	281 (26)	5820 (32)	29 063 (28)
≥60	253 (24)	7636 (42)	53 774 (52)
Estrogen receptor expression^a^			
Positive	458 (49)	12 562 (71)	85 432 (85)
Negative	468 (51)	5117 (29)	15 040 (15)
Unknown	145	642	2755

Abbreviations: GBHS, Ghana Breast Health Study; SEER 17, 17 Surveillance, Epidemiology, and End Results program registries.

^a^ Estrogen receptor expression percentages exclude unknown status.

**Table 2.** Crude and Age-Standardized Incidence Rates of Breast Cancer Cases Among Women From Ghana (GBHS) Compared With Non-Hispanic Black and Non-Hispanic White Women in the US (SEER 17), 2013-2015

Incidence assessed	Incidence rate (95% CI) per 100 000 women^a^		
	GBHS	Non-Hispanic Black, SEER 17	Non-Hispanic White, SEER 17
Overall crude	59.2 (55.7-62.8)	165.6 (163.2-168.0)	205.8 (204.6-207.1)
Overall age-standardized	84.4 (79.2-89.9)	148.5 (146.4-150.7)	152.9 (151.9-153.8)
ER-positive crude	29.4 (27.0-32.0)	117.7 (115.7-119.7)	175.0 (173.8-176.2)
ER-positive age-standardized	42.1 (38.4-46.1)	105.4 (103.6-107.3)	128.5 (127.9-129.7)
ER-negative crude	29.8 (27.4-32.4)	47.9 (46.7-49.2)	30.8 (30.3-31.3)
ER-negative age-standardized	42.3 (38.7-46.3)	43.1 (42.0-44.3)	24.0 (23.6-24.4)

Abbreviations: ER, estrogen receptor; GBHS, Ghana Breast Health Study; SEER 17, 17 Surveillance, Epidemiology, and End Results program registries.

^a^ Age-standardized incidence rates estimated using World Standard Segi 1960.^13^

Women in Ghana were diagnosed with breast cancer at earlier ages and had a higher frequency of ER-negative tumors compared with women in the US (Table 1). However, the age-standardized rates for ER-negative tumors were similar for Ghanaian (42.3 [95% CI, 38.7-46.31] per 100 000) and US non-Hispanic Black (43.1 [95% CI, 42.0-44.3] per 100 000) women, but both rates were higher than for US non-Hispanic White women (24.0 [95% CI, 23.6-24.4] per 100 000) (Table 2). In contrast, ER-positive age-standardized rates were lowest in Ghanaian women (42.1 [95% CI, 38.4-46.1] per 100 000) compared with non-Hispanic Black (105.4 [95% CI, 103.6-107.3] per 100 000) and non-Hispanic White (128.5 [95% CI, 127.9-129.7] per 100 000) women. Age-standardized breast cancer incidence rates by ER status for Ghanaian women also revealed similar rates between ER-positive (42.1 [95% CI, 38.4-46.1] per 100 000) and ER-negative (42.3 [95% CI, 38.7-46.3] per 100 000) tumors.

**Figure 1.**
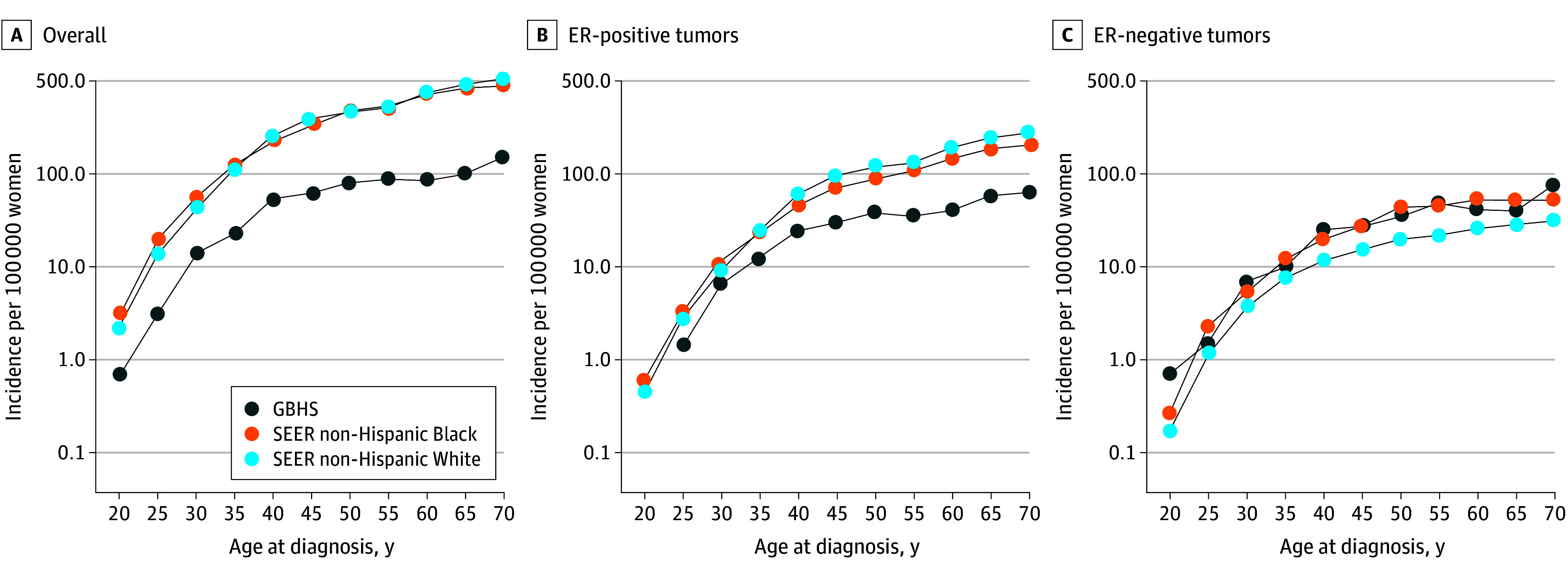
Age-Specific Breast Cancer Incidence Rate by Estrogen Receptor (ER) Status for Non-Hispanic Black and Non-Hispanic White Women From 17 Surveillance, Epidemiology, and End Results (SEER) Registries and for Women From the Ghana Breast Health Study (GBHS), 2013-2015

Age-specific incidence rates for breast cancer overall and by ER status for Ghanaian, non-Hispanic White, and non-Hispanic Black women were also estimated (Figure 1). While the overall age-specific incidence rates in Ghana were numerically lower than in the US, they showed a similar rapid increase between 20 to 50 years of age followed by a slowing of rates around 50 years of age, the approximate age at menopause. Age-specific incidence rates of breast cancer by ER status (Figure 1) showed that Ghanaian women have lower ER-positive rates at all ages compared with non-Hispanic Black and non-Hispanic White women. In contrast, ER-negative age-specific rates among Ghanaian women were comparable to those observed in non-Hispanic Black women.

**Figure 2.**
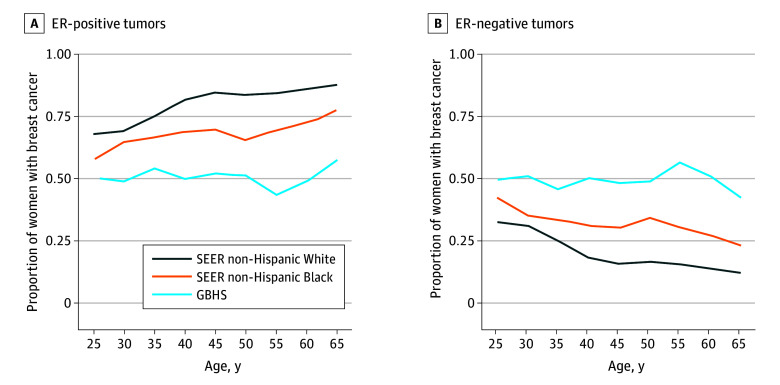
Estimated Age-Specific Proportion of Breast Cancer for Estrogen Receptor (ER)-Positive and ER-Negative Tumors Among US Non-Hispanic Black and Non-Hispanic White Women in Surveillance, Epidemiology, and End Results (SEER) Registries and for Ghanaian Women in the Ghana Breast Health Study (GBHS)

Figure 2 shows the estimated age-specific proportion of women with breast cancer in the 3 populations by ER status and age group for women 25 to 69 years of age. These data showed that Ghanaian women have the highest proportion of ER-negative breast cancers across all ages compared with non-Hispanic Black or non-Hispanic White women. The percentage of ER-negative tumors in Ghana was high at 50% and stayed relatively stable with advancing age, ranging from 42% for women 65 to 69 years of age to 57% for women 55 to 59 years of age. For non-Hispanic Black women the percentage of ER-negative tumors was lower, at about 40%, decreasing modestly with age, ranging from 27% for women 65 to 69 years of age to 42% for women 25 to 29 years of age. Both Ghanaian and non-Hispanic Black women experienced higher percentages overall compared with non-Hispanic White women, whose percentages decreased more rapidly with age, ranging from 12% for women 65 to 69 years of age to 32% for women 25 to 29 years of age.

## Discussion

The results of this cross-sectional study of breast cancer demonstrated that Ghanaian women have age-standardized rates of ER-negative breast cancer similar to those of US non-Hispanic Black women, with both groups experiencing higher rates of ER-negative breast cancer compared with US non-Hispanic White women. This is the first report of such an observation, to our knowledge. This finding was despite the evidence that Ghanaian women experienced lower age-standardized rates of overall and ER-positive breast tumors compared with US non-Hispanic Black and non-Hispanic White women.

The incidence rates stratified by ER status suggested that the lower overall incidence rates may be associated with the lower ER-positive rates experienced in Ghanaian women. The ER-positive incidence rates in Ghana may be associated in part with the limited mammography screening programs, which are more likely to detect ER-positive than ER-negative tumors.^14^ Differences in the prevalence of risk factors between the women in the US and Ghana may also be associated with the lower ER-positive rates in Ghana. In particular, reproductive factors such as younger age at first infant birth, longer duration of breastfeeding, and increased parity are inversely associated with ER-positive breast cancer risk.^8,15,16^ Nonetheless, breast cancer incidence rates are rapidly rising in Ghana and many countries possibly as a result of longer life expectancies and changing lifestyles and risk factors that have been associated with the risk of ER-positive breast cancers (eg, obesity for postmenopausal breast cancer, older age at first infant birth, and having fewer children).^15^

We observed similar age-standardized and overlapping age-specific incidence rates of ER-negative breast cancer between Ghanaian and US non-Hispanic Black women. Whether behavioral, social, environmental, or genetic factors are associated with higher incidence rates of ER-negative tumors in various populations requires additional investigation. In previous analyses, the GBHS showed that increased parity was associated with an increased risk for early-onset (<50 years) ER-negative cancers, whereas breastfeeding was inversely associated with ER-negative risk.^8^ The strengths of the GBHS are the large size and population-based design in the 2 largest cities of Ghana, standardized data collection, and quality IHC classification of ER status data for over 86% of cases.^6,8^

In the US, non-Hispanic Black women have higher incidence rates of ER-negative breast tumors compared with non-Hispanic White women after accounting for potential confounders.^17^ Temporal trends of ER-negative breast cancers in the US have shown slow declines^18^; however, non-Hispanic Black women have the highest rates across all time periods.^19^ Compared with non-Hispanic White women, non-Hispanic Black women have a higher prevalence of risk factors associated with risk of ER-negative breast cancers (eg, lower breastfeeding rates, younger age at first infant birth, and higher parity).^20^ Studies that measured individual and area levels of socioeconomic status found that socioeconomic status is not associated with ER-negative tumors, whereas these tumors were largely explained by parity and age at first infant birth; this is in contrast to socioeconomic status having an association with the incidence of ER-positive tumors.^21,22^ Higher parity has been associated with increased risk for ER-negative tumors; in contrast, breastfeeding is associated with decreased risk for ER-negative tumors.^23^ Given parity and breastfeeding prevalence rates are higher in Ghanaian women compared with US non-Hispanic Black women,^8,15,16^ it is unlikely that these reproductive factors are associated with the similar ER-negative rates of women in these countries.

The reasons for the elevated incidence rate of ER-negative breast cancer we report here among Ghanaian women are unknown. Further, our findings showed that these rates are similar to non-Hispanic Black women during the same time period, suggesting shared risk factors. The transatlantic slave trade forced the displacement of over 12 million African people, especially from West Africa,^24^ and a majority of African Americans can trace their ancestry to many current West African countries.^25^ Genetic studies estimate approximately 70% of African Americans are of West African ancestry, and a high percentage of West African ancestry has also been noted in parts of the African diaspora, including in the Caribbean and parts of Latin America.^25^ However, the percentage of African ancestry of self-identifying non-Hispanic Black individuals in the US has been shown to range anywhere from 5% to 100%.^26^ While certain markers that may be related to genetic ancestry may potentially contribute to increased ER-negative incidence rates, studies that include ancestry measures or genetic association studies are needed to evaluate this hypothesis.

In an analysis of the National Program of Cancer Registries and US Cancer Statistics of breast cancers diagnosed from 2010 through 2015, Sung et al^27^ observed that the prevalence rates of hormone-negative (estrogen- and progesterone-negative) breast cancers were higher among US-born and Western African–born Black women compared with Eastern African–born Black women, supporting that the burden of ER-negative breast cancers among Black women is not generalizable to all women of African descent. This interpretation is additionally supported by the lower frequencies of ER-negative tumors in Kenya^28^ and in South Africa^29^ as compared with Ghana.^8^

While the present study could not address ancestry measures specifically, the results are consistent with works that have measured genetic ancestry and found correlations between West African ancestry and triple-negative breast cancer^30^ as well as associations between African ancestry and gene expression profiles among different tumor subtypes.^31^ Although gaps remain in our understanding of the mechanisms underlying the reported associations between West African ancestry and triple-negative breast cancer, emerging data suggest possible underlying immunologic mechanisms^32^ or stromal disruption,^33^ biological mechanisms with implications for tumor biology as well as diagnosis and therapy.^34^

### Limitations

This study has limitations. We acknowledge the limitation of our analysis that race and ethnicity in the US is a social construct and not a biological measure, which captures a genetically heterogenous group of people. We further acknowledge that the ideal study analysis would not be based on race and ethnicity comparisons but rather on ancestry measures or genetic association studies.^35,36^ While 78% agreement between a US and Ghana laboratory for ER status is not the ideal of 90%, it is still high. In addition, this limitation in measurement error was nondifferential, suggesting that our results would tend toward the null, and rates may be even higher than reported. We anticipate that future research studies will address gaps of ancestral, environmental, and economic or social factors associated with increased risks for different breast cancer subtypes.

## Conclusions

In this cross-sectional study of Ghanaian and US non-Hispanic Black and non-Hispanic White women, we reported age-standardized and age-specific incidence rates for breast cancer overall and by ER status, revealing high ER-negative incidence rates that were similar between Ghanaian and US non-Hispanic Black women. This finding requires further investigation. Additional studies are needed to inform prevention and delineate genetic, behavioral, and social factors associated with the higher ER-negative breast cancer rates in populations with African ancestry, in which mortality rates remain among the highest globally.^13^

## Notes

Supplement 1.
Nonauthor Collaborators

Supplement 2.
Data Sharing Statement
