# Supplementary material for: Breast Cancer Incidence Rates in Ghanaian and US Black Women From 2013 Through 2015
Source: JAMA Netw Open. 2025 Oct 13;8(10):e2537160. doi: 10.1001/jamanetworkopen.2025.37160 (PMC12519306; doi:10.1001/jamanetworkopen.2025.37160)
Supplement: Supplement 1. — Nonauthor Collaborators [file jamanetwopen-e2537160-s001.pdf]

Supplemental Online Content: Nonauthor Collaborators
*First name, last name, and suffix (if applicable) are required and will appear in PubMed.

*Group Name(s): The Ghana Breast Health Study Team							
***First Name and Middle Initial(s)**	***Last Name**	***Suffix (eg, Jr, III)**	Academic Degrees	Institution	Location (city, state/province, country)	Role or Contribution, eg, chair, principal investigator	Group (if more than 1 Group listed in the byline) and/or Subgroup (eg, Steering Committee)
Nii A.	Adu-Aryee		MD	Korle Bu Teaching Hospital	Accra, Ghana	Surgeon	
Obed	Ekpedzor			Korle Bu Teaching Hospital	Accra, Ghana	Interviewer	
Angela	Kenu			Korle Bu Teaching Hospital	Accra, Ghana	Interviewer	
Victoria	Okyne			Korle Bu Teaching Hospital	Accra, Ghana	Study Manager	
Evelyn	Tay			Korle Bu Teaching Hospital	Accra, Ghana	Consultant	
Marion	Alcpaloo			Komfo Anokye Teaching Hospital	Kumasi, Ghana	Nurse	
Bernard	Arhin			Komfo Anokye Teaching Hospital	Kumasi, Ghana	Technician	
Emmanuel	Asiamah			Komfo Anokye Teaching Hospital	Kumasi, Ghana	Technician	
Isaac	Boakye			Komfo Anokye Teaching Hospital	Kumasi, Ghana	Study Manager	
Samuel	Ka-chungu			Komfo Anokye Teaching Hospital	Kumasi, Ghana	Technician	
Samuel	Amanama			Peace and Love Hospital	Kumasi, Ghana	Technician	
Emma	Abaidoo			Peace and Love Hospital	Kumasi, Ghana	Interviewer/Sample manager	
Prince	Agyapong			Peace and Love Hospital	Kumasi, Ghana	Data entry	
Thomas	Agyei			Peace and Love Hospital	Kumasi, Ghana	Technician	
Debora	Boateng-Ansong			Peace and Love Hospital	Kumasi, Ghana	Nurse	
Margaret	Frempong			Peace and Love Hospital	Kumasi, Ghana	Study Manager	
Bridget	Nortey-Mensah			Peace and Love Hospital	Kumasi, Ghana	Nurse	
Richard	Opoku			Peace and Love Hospital	Kumasi, Ghana	Technician	
Kofi	Owusu-Gyimah			Peace and Love Hospital	Kumasi, Ghana	Technician	
Lisa	Newman		MD	Weill Cornell School of Medicine	New York, NY	Surgeon	
Stephen	Hewitt		MD	National Cancer Institute	Bethesda, MD	Pathologist	
Petra	Lenz		MD	National Cancer Institute	Bethesda, MD	Pathologist	
Maire	Duggan		MD	Cumming School of Medicine, Univ	Calgary, Canada	Pathologist	

Supplemental Online Content: Nonauthor Collaborators
*First name, last name, and suffix (if applicable) are required and will appear in PubMed.

***First Name and Middle Initial(s)**	***Last Name**	***Suffix (eg, Jr, III)**	Academic Degrees	Institution	Location (city, state/province, country)	Role or Contribution, eg, chair, principal investigator	Group (if more than 1 Group listed in the byline) and/or Subgroup (eg, Steering Committee)
Ricardo	Diaz			Westat, Inc.	Rockville, MD	Field Study support	
Shelley	Niwa			Westat, Inc.	Rockville, MD	Data support	
Usha	Singh			Westat, Inc.	Rockville, MD	Field Study support	
Ann	Truelove			Westat, Inc.	Rockville, MD	Field Study support	
Michelle	Brotzman			Westat, Inc.	Rockville, MD	Study support and data support	
