# Supplementary material for: Breast Cancer Incidence Rates in Ghanaian and US Black Women From 2013 Through 2015
Source: JAMA Netw Open. 2025 Oct 13;8(10):e2537160. doi: 10.1001/jamanetworkopen.2025.37160 (PMC12519306; doi:10.1001/jamanetworkopen.2025.37160)
Supplement: Supplement 2. — Data Sharing Statement [file jamanetwopen-e2537160-s002.pdf]

Data Sharing Statement

Davis-Lynn. Breast Cancer Incidence Rates in Ghanaian and US Black Women From 2013 Through 2015. *JAMA Netw Open*. Published October 13, 2025.
doi:10.1001/jamanetworkopen.2025.37160

Data
Data available: Yes
Data types: Deidentified participant data, Data dictionary
How to access data: jonine.figueroa@nih.gov
When available: With publication

Supporting Documents
Document types: Statistical/analytic code
How to access documents: jonine.figueroa@nih.gov
When available: With publication

Additional Information
Who can access the data: We will place them in a website either github or an NIH designated website.
Types of analyses: Counts of cases and weights for age-standardized and age-specific rates
Mechanisms of data availability: As these are counts data we will provide them in 5 year age groups with restrictions to informed consent form restricted studies related to adult diseases
